# Facile aerobic construction of iron based ferromagnetic nanostructures by a novel microbial nanofactory isolated from tropical freshwater wetlands

**DOI:** 10.1186/s12934-017-0789-3

**Published:** 2017-10-11

**Authors:** Patricia Jayshree Jacob, Mas Jaffri Masarudin, Mohd Zobir Hussein, Raha Abdul Rahim

**Affiliations:** 1 Department of Cell and Molecular Biology, Faculty of Biotechnology and Biomolecular Sciences, Universiti Putra Malaysia, 43400 UPM Serdang, Selangor Malaysia; 2 Institute of Biosciences, Universiti Putra Malaysia, 43400 UPM Serdang, Selangor Malaysia; 3 Institute of Advanced Technology, Universiti Putra Malaysia, 43400 UPM Serdang, Selangor Malaysia

**Keywords:** Iron oxide nanoparticles, Ferromagnetic nanoparticles, Aerobic bacteria, Extracellular biosynthesis, Melanin mediated, Tropical freshwater ecosystems

## Abstract

**Background:**

Iron based ferromagnetic nanoparticles (IONP) have found a wide range of application in microelectronics, chemotherapeutic cell targeting, and as contrast enhancers in MRI. As such, the design of well-defined monodisperse IONPs is crucial to ensure effectiveness in these applications. Although these nanostructures are currently manufactured using chemical and physical processes, these methods are not environmentally conducive and weigh heavily on energy and outlays. Certain microorganisms have the innate ability to reduce metallic ions in aqueous solution and generate nano-sized IONP’s with narrow size distribution. Harnessing this potential is a way forward in constructing microbial nanofactories, capable of churning out high yields of well-defined IONP’s with physico-chemical characteristics on par with the synthetically produced ones.

**Results:**

In this work, we report the molecular characterization of an actinomycetes, isolated from tropical freshwater wetlands sediments, that demonstrated rapid aerobic extracellular reduction of ferric ions to generate iron based nanoparticles. Characterization of these nanoparticles was carried out using Field Emission Scanning Electron Microscope with energy dispersive X-ray spectroscopy (FESEM–EDX), Field Emission Transmission Electron Microscope (FETEM), Ultraviolet–Visible (UV–Vis) Spectrophotometer, dynamic light scattering (DLS) and Fourier transform infrared spectroscopy (FTIR). This process was carried out at room temperature and humidity and under aerobic conditions and could be developed as an environmental friendly, cost effective bioprocess for the production of IONP’s.

**Conclusion:**

While it is undeniable that iron reducing microorganisms confer a largely untapped resource as potent nanofactories, these bioprocesses are largely anaerobic and hampered by the low reaction rates, highly stringent microbial cultural conditions and polydispersed nanostructures. In this work, the novel isolate demonstrated rapid, aerobic reduction of ferric ions in its extracellular matrix, resulting in IONPs of relatively narrow size distribution which are easily extracted and purified without the need for convoluted procedures. It is therefore hoped that this isolate could be potentially developed as an effective nanofactory in the future.

## Background

Ferric based magnetic nanoparticles (IONP) have drawn a great deal of interest due to their versatility for applications in microelectronics, environmental remediation and theranostics [1–4]. The central role of IONP in bio-medicine as high resolution contrast agents in magnetic resonance imaging (MRI) [5] and in magnetically guided chemotherapeutic delivery to malignant cells via hyperthermia [6] is undeniable. In addition to its pertinent role in targeting and destroying malignant cells, IONP’s can be engineered for in vivo applications such as cellular therapy [7], bioseparation [8] and immunoassay [9]. Multifunctional IONP’s have also been used as nanoadsorbents in water treatment and purification and as sensitive biosensors [10] that track down formidable pollutants in aqueous solutions.

Due to its widespread application potential, the synthesis of well-defined ferromagnetic IONP’s has emerged as a widely investigated research niche. Currently, most synthesis protocols rely heavily on chemical methods including co-precipitation [11], thermal decomposition [12], atomic layer deposition, [13] and sol–gel processes [14], or physical protocols such as ball milling [15], electron beam evaporation [16], radio frequency (RF) sputtering [17] or sonochemical synthesis [18]. Although these methods produce predictable outcomes, they require rigorous adherence to stringent protocols and entail a high cost and energy investment. Additionally, potent chemicals such as dialkylamide [19], octadecene or oleylamine [20] are often used as capping agents, solvents or stabilizers which are hazardous and leave noxious environmental residues [21]. Furthermore, producing biocompatible nanostructures with uniform morphology poses a surmountable challenge in large scale production.

These shortcomings have therefore led to the pursuit of alternative routes in nanoparticle synthesis. Biosynthesis routes unravel the innate ability of sustainable bio-systems such as plants and algae [22], bacteria [23] and fungi [24] to generate large amounts of uniform, mono-dispersed metallic nanoparticles and have emerged as a promising preference towards the design of environmentally sound, biocompatible IONPs. In general, the biosynthesis of metallic nanoparticles employs two mechanisms—bioreduction [25] which reduces metal ions in salt solution to stable nanostructures using dissimilatory metal reduction [26] and intracellular biomineralization where iron is imported into the cell and well-defined IONP crystals such as magnetite are constructed in organelles known as magnetosomes [27].

Bacteria have demonstrated tremendous potential as emerging nanofactories, conferring the cellular mechanisms to accumulate high concentrations of metallic ions and possessing the enzymatic machinery to catalyze the reduction of metallic ions in ferrofluids to nano-sized iron crystals at ambient temperature and pressure [28]. Their occurrence in extreme habitats denotes their unique enzymatic machinery which could substitute the need for potent solvents or catalysts in nanoparticle fabrication. Bharde [28], Byrne et al. [29] postulated that fine-tuning specific process parameters such as incubation time, biomass and precursor concentration could generate stable, uniform-sized, well defined nanostructures without the need for synthetic capping agents and stabilizers. Finally, bioprocesses can be easily in scaled-up for cost-effective large scale production [30], without leaving trails of noxious residues and byproducts.

Although intracellular biomineralization generates mono-dispersed and intricately designed nanomagnets with uniform particle dimensions [31], extracting and purifying these intracellular nanocrystals downstream and producing sufficient biomass of industrial proportions is challenging. The downstream process of extracting and purifying nanoparticles generated using Fe(III)-reducing bacteria such as Shewanella and Theroanaerobacter [32] through extracellular reduction of ferric ions in aqueous solution is less challenging and results is better yield. Unfortunately, most Fe reducing bacteria are anaerobic requiring anoxic growth conditions in bioreactors and long periods of incubation for growth in well formulated media [33]. Therefore, a relentless hunt for aerobic microorganisms capable of rapid extracellular production of IONPs capable of being developed as efficient nanofactories for industrial scale production is essential.

The tropical aquatic environment offers a species rich environment for the bioprospection of highly potent microorganisms capable of generating attractive bioactive ingredients for commercial use. This is largely due to its conducive environmental conditions in soil fertility, water availability and temperature consistency that enable a large array of organisms to thrive comfortably in these well-carved out niches. Unfortunately, the tropical aquatic ecosystem is largely unexplored and few reports exist on microbial factories originating from these freshwater or marine ecosystems, thereby necessitating an expedition into this ocean of opportunity.

We describe in this study, a novel pigmented aerobic actinomycete isolated from freshwater sediments of a tropical rainforest in Peninsular Malaysia which rapidly reduced metallic iron precursors in aqueous solution to generate IONP’s in considerable quantities under aerobic conditions and ambient temperature. Through this investigation, we established that this isolate has the potential to be further developed as a viable green nano-factory capable of large scale sustainable production of well-defined IONPs.

## Methods

### Sampling and isolation of aquatic microorganisms

Samples were collected from freshwater wetlands sediments at coordinates 2.8289^o^N, 101.8236^o^E in a tropical rainforest at Peninsular Malaysia. These sediment samples were collected from the river sediments at a depth of 15–20 cm from the surface using a sterile cork borer, transferred to sterile petri dishes and stored at 4 °C.

### Isolation of bacteria

1 g of sediments from the samples that were collected were suspended in 10 ml sterilized river water and vortexed for 1 min. 1 ml of this suspension was removed and placed in a boiling tube with 9 ml sterilized river water. This process was repeated until a dilution factor of 10^−5^. 50 µl of suspension from this dilution was removed and spread plated on Nutrient Agar (Peptone 5 g/l, sodium chloride 5 g/l, beef extract 1.5 g/l, yeast extract 1.5 g/l and Agar 15 g) (NA) (Himedia India) and incubated at room temperature, 28 °C for 5 days. The colonies that survived under laboratory conditions and demonstrated unique morphology were picked and streaked on NA plates before screening for IONP production.

### Screening isolates for extracellular biosynthesis of IONP

To screen for the extracellular biosynthesis of IONP, single colonies of all isolated bacteria were inoculated in 5 ml freshly prepared sterile Nutrient Broth (NB) (HiMedia, India) and incubated at room temperature for 24 h in a rotary shaker (Lab Companion S1-300) at 120 rpm. This seed culture was then transferred into a Falcon tube with 30 ml NB and incubated at 30 °C for 48 h at 120 rpm. The cells were then harvested by centrifugation (6000 rpm, 28 °C, 15 min) using a Hettich Universal 16 Benchtop Centrifuge (1624 Rotor) and the supernatant transferred to another tube. Equal volumes of filter sterilized IONP precursor, 1 mM FeCl_3_·6H_2_O (QRec, Italy) was added to the supernatant and incubated in the dark at 120 rpm at 30 °C for 48 h. This procedure was repeated using an equal volume of another precursor, 1 mM FeSO_4_·7H_2_O and observed. The formation of iron oxide nanoparticles was indicated through the change of the color from yellow to dark brown, or black. The isolated bacteria that demonstrated this color change was then characterized using 16S rRNA sequencing protocols [34] for identification.

### Molecular identification of positive isolate using 16S rRNA sequencing

Molecular identification of the positive isolate was carried out using 16S rRNA sequencing [34]. Bacterial 16S rDna was amplified using 1492R (5′TACGGYTACCTTGTTACGACTT3′) and 27F (5′AGAGTTTGATCMTGGCTCAG 3′) universal primers. The total volume of 25 µl PCR reaction mixture consisted of 0.5 µl bacterial DNA (1 ng/ml), 10 pmol of each forward and reverse primers, 0.5 µl of deoxynucleotides triphosphates (dNTPs, 400 µM each), 0.75 µl Taq DNA polymerase and PCR buffer (contents). The PCR was 0.5 µl DNA (1 ng/ml) from crude bacterial lysate, 0.5 µl (10 pmol) of each primer, 0.5 µl of each deoxynucleotides triphosphates (dNTP’s) (400 µM), 0.8 µl Taq DNA polymerase, 5 µl 10× PCR buffer and sterile distilled water. The PCR was performed as follows: 1 cycle (95 °C for 5 min) for initial denaturation; 30 cycles (95 °C for 45 s; 51 °C for 15 s; 72 °C for 2 min) for annealing and extension, and 1 cycle (72 °C for 10 min) for final extension of the amplified DNA. Purified PCR products were subsequently sequenced with 518F and 800R primers using the BigDye^®^ Terminator v3.1 Cycle Sequencing Kit (Applied Biosystems). The sequenced DNA was then compared for percent similarity in a BLAST sequence similarity search on the NCBI website [34].

### Characterization of iron oxide nanoparticles

The formation of IONPs in bacterial supernatant was initially examined by measuring the optical density of the samples using a UV–Visible spectrophotometer (Shimadzu UV-160, Japan) at wavelengths ranging from 300 to 600 nm. For FESEM–EDX characterization, the bacterial supernatant containing IONP’s was centrifuged at 15,000 rpm for 45 min to separate the nanoparticles from the liquid culture. The pellet containing IONP was re-suspended in 2 ml deionized sterile water and filter sterilized using a 45 µm filter. 300 µl of this suspension was drop coated on aluminium stubs and air-dried for 2 days. The sample was then gold-coated and characterized using FESEM (FEI Nova NanoSEM 230 FE-SEM). To determine the elemental composition of the sample, EDX spectroscopy with an imaging resolution of 1 nm at 15 kV was used together with FESEM. Further morphological characterization was carried out using a Field Emission Transmission Electron Microscope (TEM) Model JEM-2100F (JEOL, Munich, Germany) operating at beam strength of 200 kV. The IONP’s suspension was first dispersed using an ultrasonic bath, and then deposited on a copper grid and air-dried overnight. To further verify the size distribution of the biosynthesized IONP’s, dynamic light scattering (DLS) was performed using the Zetasizer (Malvern Instruments, USA), operating at a scattering angle of 173° was used. In this method, when the incident ray from a light beam impinges on the IONP’s in colloidal solution, the direction of the light beam is scattered based on the size of the nanoparticle [35]. This gives us an accurate postulation of the size distribution and polydispersity of the biosynthesized IONPs. Finally, the surface chemistry of the biosynthesized iron based nanoparticles was analyzed by Fourier-Transform Infrared Spectroscopy using a Thermo Nicolet 6700 FTIR.

## Results

### Isolation and screening of aquatic microorganisms for extracellular biosynthesis of IONP

A total of 24 bacterial strains that survived under laboratory conditions and demonstrated unique morphology and pigmentation were recovered from the riverbed sediment samples. The morphological characteristics of the lab survived bacteria are depicted in Table 1.

**Table 1:** Morphological characteristics of the tropical riverbed bacterial isolates

No.	Isolate	Colony morphology	No.	Isolate	Colony morphology
1	MS1	Circular, raised, yellow	*13*	MS13	Irregular, concave, white
2	MS2	Creamish, umbonate,	*14*	MS14	Irregular, convex, pale yellow
3	MS3	Circular, flat, milky white	*15*	MS15	Circular, raised, pale peach
4	MS4	Circular, raised, peach	*16*	MS16	Circular, flat, red
5	MS5	Circular, flat, orange	*17*	MS17	Irregular, flat orange
6	MS6	Circular, raised green	*18*	MS18	Irregular, raised, yellow
7	MS7	Circular, flat, pale yellow	*19*	MS19	Irregular, white, convex
8	MS8	Irregular, raised, yellow	*20*	MS20	Irregular, creamish, flat
9	MS9	Circular, raised, white	*21*	MS21	Circular, convex, brown
10	MS10	Circular, raised, orange	*22*	MS22	Circular, raised, dark yellow
11	MS11	Circular, convex, purple	*23*	MS23	Threadlike projections, white
12	MS12	Circular, convex yellow	*24*	MS24	Circular, convex, black

All positive bacterial isolates formed colonies on NA and were stored at room temperature

**Fig. 1:**
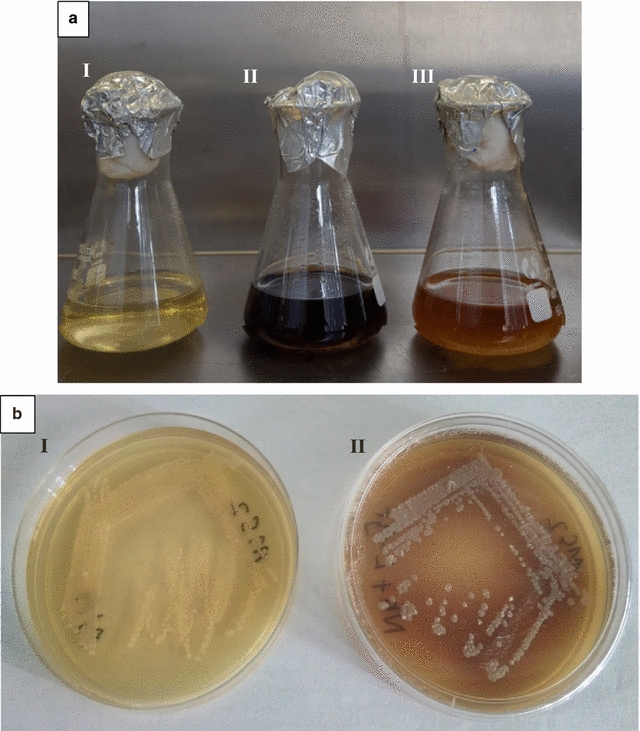
**a** Positive isolate MS2 (i) NB without bacterial culture (ii) MS2 in LB when added culture with FeCL_3_·6H_2_O (iii) MS2 without precursor. Positive MS2 bacterial isolates formed. **b** Cream colored white colonies on regular NA as compared to diffusible brown pigmentation on NA supplemented with 5 mg/L l-tyrosine

When challenged with the IONP precursors, 1 mM FeCl_3_·6H_2_O and 1 mM FeSO_4_·7H_2_O, only one strain, MS2, demonstrated the reduction of ferric ions in aqueous solution to generate IONP’s as observed in a colorimetric change in the liquid culture from yellow to dark brown within 5 min after adding the precursors (Fig. 1a). This colorimetric change was a preliminary indication of a positive outcome. MS2 formed tough, cream-colored, leathery colonies which formed powdery spore-like structures on NA without any l-tyrosine and diffusible brownish black pigmentation when plated on l-tyrosine supplemented NA as depicted in Fig. 1b.

### Molecular identification of positive isolates using 16S rRNA sequencing

Molecular identification of the positive isolate, MS2 was carried out using16S rRNA sequencing, based on the percentage of similarities in the highly conserved hypervariable regions of 16S rRNA gene sequences.

**Fig. 2:**
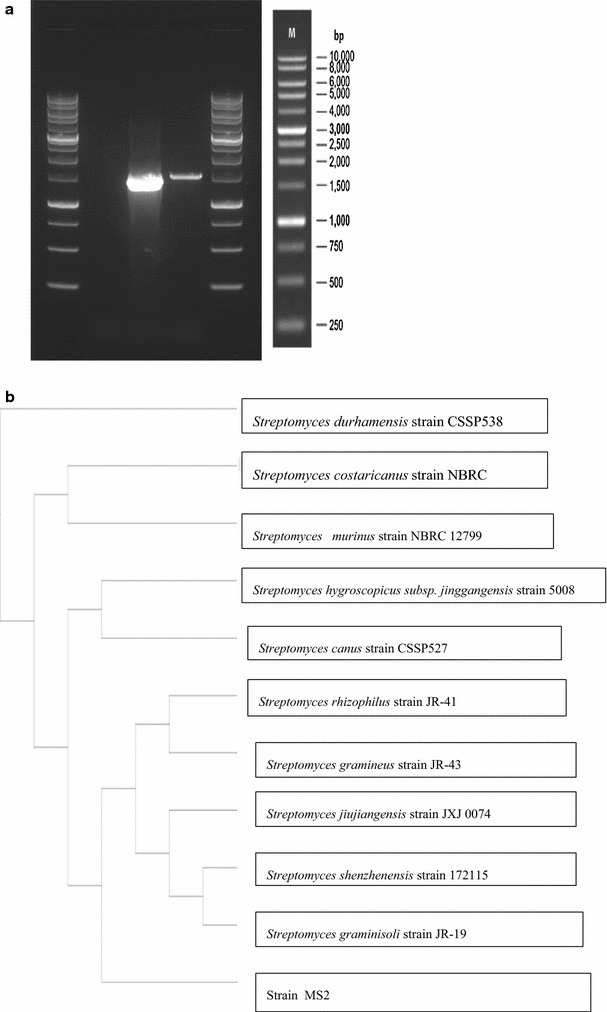
**a** Gel image of the PCR amplified 16S rRNA sequence depicted as a single band on the agarose gel. **b** Phylogenetic tree constructed based on comparison between 10 sequences of high percentage similarities

Figure 2a shows the results of the PCR amplified 16S rRNA gene depicted as a single band on the agarose gel. The PCR product was purified and sequenced and subjected to a BLAST query as depicted in Table 2. MS2 sequence showed maximum homology (99%) with 3 species namely *Streptomyces jiujiangensis* strain JXJ 0074 (Genbank Accession No. NR 1257061), *Streptomyces rhizophilus* strain JR-41 (Genbank Accession No. NR 125578 1) and *Streptomyces gramineus* strain JR-43 (Genbank Accession No. NR 109017 1) with a high score and zero e-value. A phylogenetic tree (Fig. 2b) was constructed using the Neighbor Joining (Unrooted Tree) NCBI Blast Tree Method where the branches of the phylogenetic tree is based on the distance originating from an unrooted tree which depict the similarities within sequences. Phylogenetic analysis revealed Strain MS2 as belonging to the *Streptomyces* genera due to the high percentage of similarities with the strains in this tree but possessing exclusive features to warrant a branch of its own.

**Table 2:** Results showing percentage similarities against NCBI 16S ribosomal RNA sequences (Bacteria only)

Description	Max score	Total score	Query cover (%)	E value	Ident (%)	Accession
*Streptomyces jiujiangensis strain JXJ 0074*	2473	2473	100	0.0	99	NR_125706.1
*Streptomyces rhizophilus strain JR*-*41*	2459	2459	99	0.0	99	NR_125578.1
*Streptomyces gramineus strain JR*-*43*	2452	2452	99	0.0	99	NR_109017.1
*Streptomyces shenzhenensis strain 172115*	2450	2450	100	0.0	98	NR_118018.1
*Streptomyces hygroscopicus jinggangensis 5008*	2448	2448	100	0.0	98	NR_074563.1
*Streptomyces canus strain CSSP527*	2444	2444	100	0.0	98	NR_043347.1
*Streptomyces graminisoli strain JR*-*19*	2443	2443	99	0.0	98	NR_125577.1
*Streptomyces costaricanus strain NBRC 100773*	2441	2441	99	0.0	98	NR_041414.1
*Streptomyces murinus strain NBRC 12799*	2439	2439	99	0.0	98	NR_041072.1
*Streptomyces durhamensis strain CSSP538*	2439	2439	100	0.0	98	NR_043352.1

### Morphological characterization of IONP using FESEM–EDX and FETEM

**Fig. 3:**
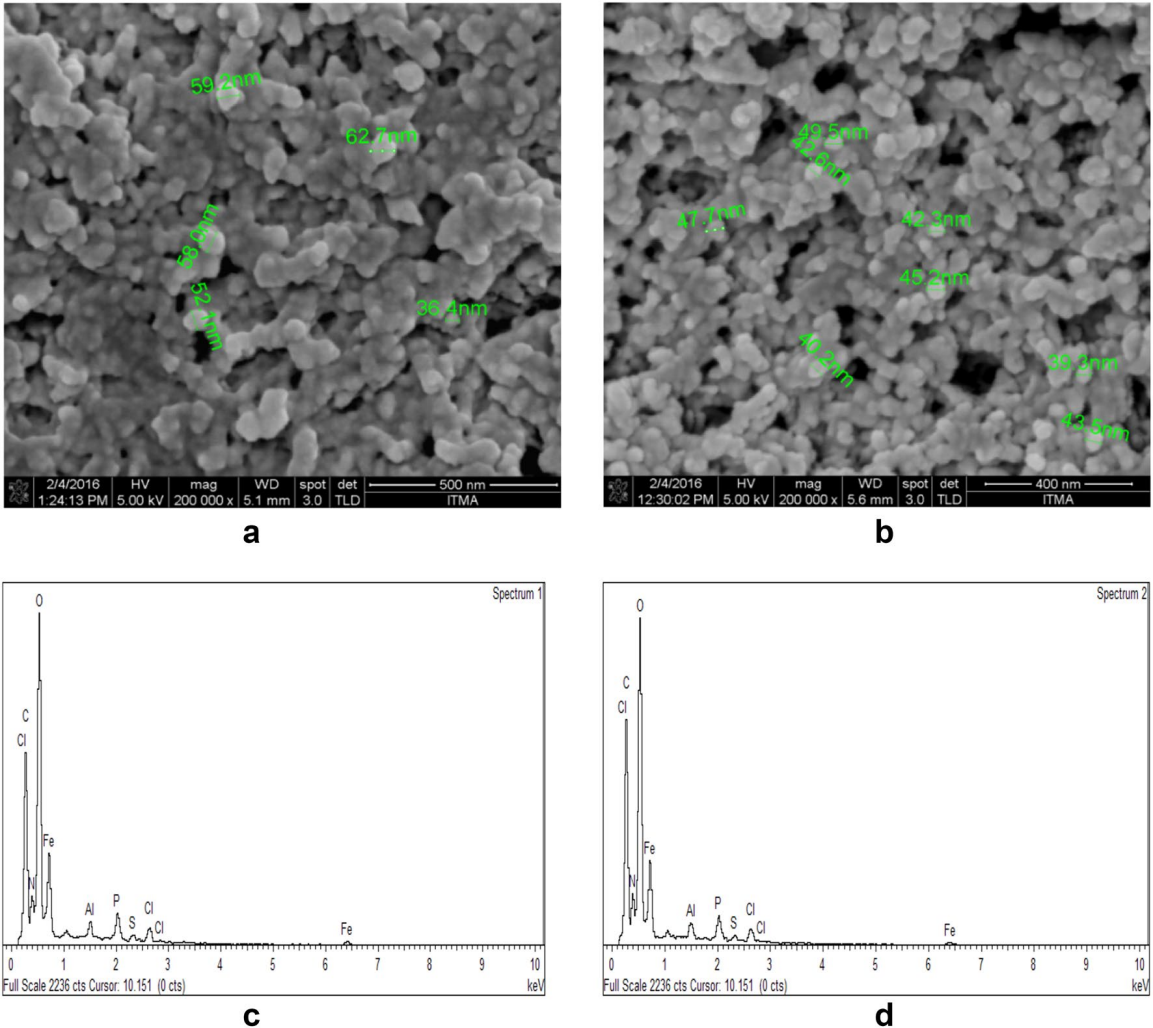
**a**, **b** FESEM micrographs and **c**, **d** EDX profiles of biosynthesized IONP using FeCl_3_·6H_2_O and FeSO_4_·7H_2_O as precursors respectively

Field Emission Scanning Electron Microscope-Energy Dispersive Spectrometer (FESEM–EDS) was used in surface morphology and dimensional characterization of the biosynthesized IONP’s. The sharp probing beams emitted by the field emission cathode in a FESEM are able to provide excellent spatial resolution with minimal sample intrusion in the morphological characterization, surface chemistry and size uniformity of IONP’s. FESEM images (Fig. 3a, b) demonstrated that MS2 was able to reduce ferric ions in aqueous solutions and generate well-defined, fairly uniform-sized iron based nanoparticles in the size range of 30–60 nm. The appearance of particles as aggregates in the electron microscopy images was thought to be a consequence from the drying process during sample preparation and not a physical representation of the nanoparticles synthesized, a phenomenon that was previously reported [36–38]. EDX is an energy dispersive X-ray detector which analyzes and determines the elemental composition of component particles on the surface of the IONP’s. EDX profiles (Fig. 3c, d) based on percentage composition of elements present (Table 3) confirmed the presence of Fe (35.47%) and oxygen (29.94%). Other elements C, N and P represented in the EDX profiles could be present due to bacterial debris, while the presence of Al could be caused by the Al grids used throughout sample preparation.

**Table 3:** Elemental breakdown of biosynthesized MNP based on EDX profiles

Element	C	N	O	Al	P	S	Fe	Cl
Weight%	22.41	6.75	29.94	0.86	2.24	0.55	35.47	1.77
Atomic%	37.13	9.59	37.23	0.64	1.44	0.34	12.64	0.99

**Fig. 4:**
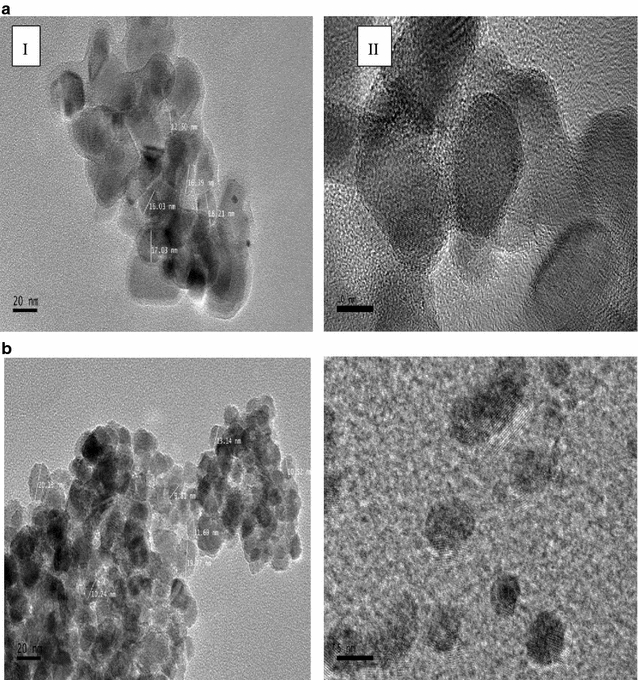
**a** Field emission TEM images of IONP’s formed using FeCl_3_·6H_2_O as precursor at (I) 100,000× magnification and (II) crystal lattice structure of IONP at 300,000×. **b** IONP synthesized using FeSO_4_·7H_2_O as precursor at (I) 100,000× magnification and (II) IONP lattice structure (600,000×)

From the Field Emission TEM images in Fig. 4a, b, the agglomeration of the biosynthesized IONP’s was evident as perceived in both the images, possibly caused by the nanoparticles still being suspended in the bacterial extracellular matrix in the supernatant. The agglomeration of these nanoparticles made it difficult to obtain individual particles for measurement but close observation revealed that the IONP’s were irregular shaped with a narrow size range between 10 and 20 nm. Through close observation of the crystal lattice structure at higher magnification (Fig. 4a(ii)), the mono- crystalline structure of the biosynthesized IONPs could be verified.

### Characterization of IONP using UV–Vis spectroscopy

**Fig. 5:**
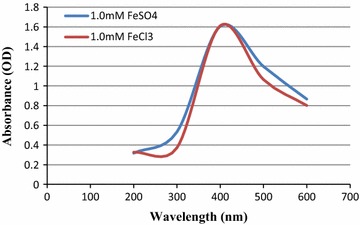
UV-Vis spectrum of the biosynthesized IONP using FeSO_4_·7H_2_0 and FeCl_3_·6H_2_O as precursors

The presence of biosynthesized IONP’s in the bacterial supernatant was determined based on characteristic colorimetric changes and absorption band in UV–Visible Spectrophotometry. In this study, a rapid color change from yellowish brown to black, distinctive to IONP’s, was observed in the bacterial supernatant upon the addition of the IONP precursors. The UV–Vis spectrum reflects the surface plasmon resonance (SPR) of nanomaterials, a phenomenon attributed to a compilation of different oscillation moments of free moving surface electrons in response to an electromagnetic field [39]. Here, metallic nanoparticles in colloidal aqueous solution display a characteristic absorption band in the UV–Vis spectrum contributed by the dielectric constant between the solvent and surface-adsorbed species [40]. In this study, an absorption peak of 370–400 nm (Fig. 5) is observed, coinciding with the absorption spectra of magnetite (Fe_3_O_4_) superparamagnetic nanoparticles as reported by Rahman et al. [41]. This absorption peak (Fig. 4) appears as a narrow band suggesting that the biosynthesized IONPs could be monodispersed and spherical in shape, as spherical nanoparticles, as postulated by Mie [42] are expected to give a singular, narrow peak in the absorption spectra whereas anisotropic nanoparticles could give rise to multiple bands.

### Size distribution measurements of biosynthesized IONPs using dynamic light scattering

Dynamic light scattering (DLS) was used to ascertain the average hydrodynamic diameter [43] and monodispersity of the IONP’s, with the underlying concept being the measurement of the scattering of a light beam when it strikes the IONP’s in colloidal solution. This technique has been widely used for determining the size of metallic nanoparticles in liquid phase and its precision is affected by the concentration of suspension, angle of scattering and anisotropy of nanoparticles [44]. The average percentage intensity is calculated based on 3 replicate measurements.

**Fig. 6:**
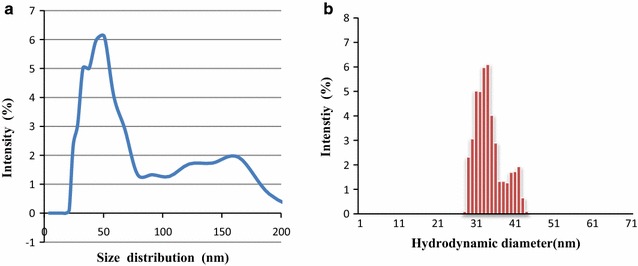
**a** Depicting size distribution comparing to average light scattering intensity and **b** representing hydrodynamic diameter of biosynthesized IONP

Figure 6a shows the particle size distribution of the IONP’s in colloidal solution From Fig. 6a, the average hydrodynamic particle size distribution of the IONP’s fall within the range of 20 nm to over 240 nm with the highest intensity of light scattering being within 30–50 nm in range as depicted in the FESEM images. The average polydispersity index (PDi) was calculated at 0.394.

Table 4 depicts the zeta average, polydispersity index and percentage intensity of the nanoparticles in solution. Zeta average values provide the average hydrodynamic size of particles in solution, which is between 50 and 60 nm, coinciding with the particle size depicted in the FESEM images. The polydispersity index which is measure of the IONP size distribution here is calculated to be an average of 0.52, which is an indication of the narrow size distribution of the manufactured nanoparticles.

**Table 4:** The Zeta potential, polydispersity index and percentage intensity of the IONPs

Runs	Zeta average (mV)	Polydispersity index (PDi)	Intensity (%)
1	50.99	0.44	42.45 (70.2%), 119 (19%)
2	60.33	0.900	151 (61.4%), 17.8 (27.6%)
3	53.54	0.210	71.29 (98%)

### Fourier transform infrared spectrocopy (FTIR)

**Fig. 7:**
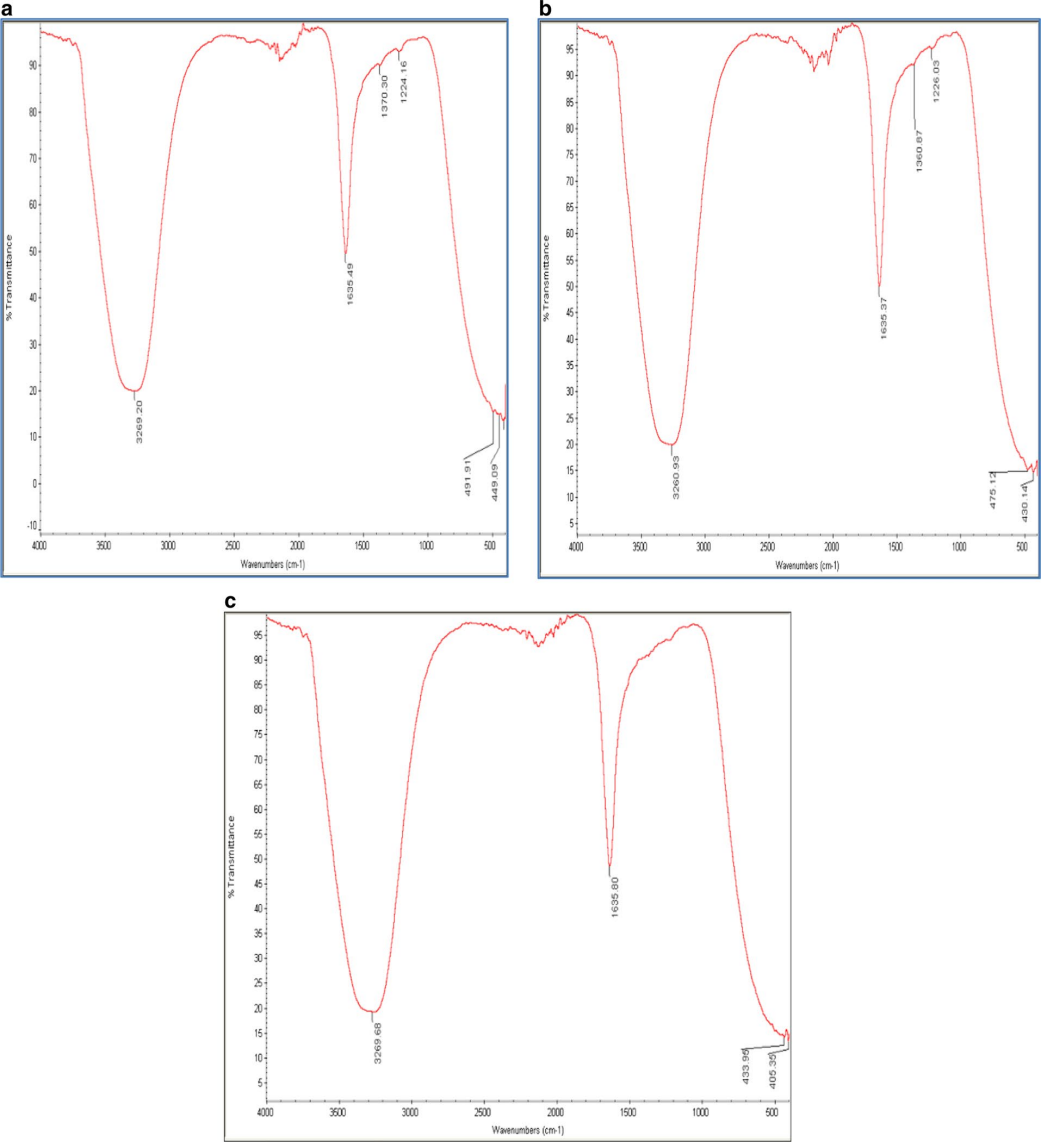
FTIR profile of IONP’s generated with **a** FeCl3·6H2O, **b** FeSO4·7H2O compared with **c** control supernatant without a precursor

Fourier transform infrared spectrocopy profiles of both precursor added culture supernatant and control are depicted in Fig. 7. Figure 7a, b shows the FTIR spectrum of the colloidal IONP’s synthesized with the precursors FeCL_3_·6H_2_O and FeSO_4_·7H_2_O respectively. The bands at 3260–3270 correspond to the O–H stretching and bending. The formation of IONP’s were depicted in the characteristic peaks between 400 and 600 cm^−1^ [44] for biosynthesized IONP’s where the bands are shifted to lower frequencies due to the presence of the C=O and –OH bands. The band found at 449 cm^−1^ (Fig. 7a) and 430 cm^−1^ (Fig. 7b) could be assigned to octahedral-metal stretching of Fe–O [44], confirming the octahedral crystalline shape of the biosynthesized IONP’s. When compared to the control FTIR profile (Fig. 7c) without the addition of any precursors, both FTIR profiles (Fig. 7a, b) showed additional peaks in 1350–1370 cm^−1^ originating from the C–N stretch vibration of the aromatic amine I and amine II and 1224–1227 cm^−1^ corresponding to C–H stretching and O–H deformation of carboxyl groups and to the N–H bond of amide II [45]. This could be attributed to the phenolic groups of the pigment which contributes to reduction of the ferric ions to IONP’s.

## Discussion

### Screening and isolation of a potential IONP generating microbial nanofactory

The tropical freshwater ecosystems represent among the richest biodiversity in the world, wherein lie great mysteries waiting to be unraveled. Microorganisms that thrive in this environment demonstrate adaptation mechanisms such as biosorption, biomineralization, extracellular sequestration and chelation in coping with this heavy metal laden environment [46]. These adaptive alternative biochemical pathways create the platform for the generation of metallic nanoparticles through intracellular biomineralization or extracellular bioreduction metallic ions in aqueous solution.

Iron based ferromagnetic nanoparticles have attracted much attention due to its versatility in pertinent applications. Magnetotactic bacteria produce uniform sized, well-defined magnetite nanoparticles in their magnetosomes but harvesting these IONP’s involve scrupulous downstream extraction and purification processes. Extracellular biosynthesis has the potential of generating large volume of IONP’s but the process is time-consuming, requires stringent culture conditions for anaerobic bacteria and IONP’s formed have poor morphology. It is therefore imperative that for a microorganism to be developed into a IONP nanofactory, it would demonstrate rapid generation of uniformed sized nanoparticles in large volumes at ambient conditions.

The isolates screened in this study originated from both the freshwater and marine sediment samples. As these environments are rich in mineral resources, wide arrays of microorganisms with unique morphological features were isolated. Most marine bacteria demonstrated unique pigmentation that aids in the survival of the microorganism in a halophytic environment [47] but none of these isolates demonstrated the ability to reduce Fe^3+^ ions to form IONP. Although the marine environment is home to iron reducing bacteria, they are usually found in deep sea sediments where they thrive as obligate anaerobes.

The positive isolate, MS2 originated from the tropical freshwater environment of a riverbed soil sediments. It shouldn’t be overlooked that soil sediment samples from a tropical aquatic environment comprise rich biodiversity due to its consistent weather conditions and rich nutrient composition making it a recommendable site in microbial bioprospection of industrial potential.

The positive isolate MS2 demonstrated rapid reduction of Fe^3+^ ions in aqueous solution at ambient temperature with minimal media requirements. This positive isolate grew under aerobic conditions and took less than 48 h to appear on basal NA. Molecular identification of this Gram positive isolate traced it to the *Streptomyces* genera and on l-tyrosine enriched media, a diffusible brown pigment, possibly melanin, was observed. Melanin producing microorganisms have generated silver and gold nanoparticles [48] but this is the first attempt in using melanin pigmented bacteria to produce IONP’s. Pyomelanin, a derivative of melanin, has been proven to confer ferric reductase activity in *Legionella pneumophilia* when confronted with Fe^3+^ in surrounding media, enabling iron acquisition and assimilation through the cellular membrane [49], postulating that this could be a similar mechanism used in this bioprocess as evidenced with similar pigmentation in MS2.

FESEM images captured the morphological structure of biosynthesized IONP’s with narrow size distribution between 35 and 50 nm. The appearance of IONP’s as micro-aggregates could be due to the extreme heat and drying during sample preparation that encourages the particles to be drawn to the other as an aggregate of particles. In order to determine the elemental composition of the crystalline nanostructures, EDX analysis was carried out that demonstrates the atomic percentage of the elements present in the solution. The predominant peaks as depicted in this analysis were Fe and O suggesting the presence of IONP and C which could originate from extracellular bacterial matrix in the supernatant. The TEM images confirm the agglomeration depicted in the FESEM images possibly caused by high surface energies of raw IONPs due to the high surface area per volume ratio of nano-sized particles. This agglomeration could be caused by the amorphous layer of bacterial extracellular matrix which may contain stabilizing proteins that protect IONP structure.

There is, however, a significant difference in the sizes of nanoparticles measured using FESEM (between 36.4 and 62.7 nm) as compared to the field emission TEM (from 9.81 to 20.81 nm). This could be because TEM has higher resolution than SEM, allowing individual particles to be measured separately and accurately compared to FESEM images which may be measuring a group of particles due to lower resolution. Also, samples for FESEM analysis have been gold coated, adding to the particle size compared to TEM samples which are not coated. Furthermore, biosynthesized IONPs are still covered in extracellular polymeric matrix which are visible under FESEM but not in TEM where electron beams can penetrate through the polymeric matrix to reveal the actual sizes of embedded individual particles.

Nanoparticles have optical properties that are very sensitive to size, shape, agglomeration, and concentration changes. The unique optical properties of metal nanoparticles are a consequence of the collective oscillations of surface conduction electrons when excited by electromagnetic radiation, called surface plasmon resonances (SPPR) [40]. Those changes have an influence on the refractive index next to the nanoparticles surface. Using this phenomena, IONP’s could be characterized using UV–Vis spectroscopy. From the absorbance spectra (Fig. 5), the formation of IONP’s were depicted at its characteristic peak of 370–400 nm. The absorption maxima is very similar to β-Fe_2_O_3_ as reported by Cornell and Schwertmann [1, 36–38] but further investigation is needed to determine the exact derivative of the iron based nanoparticle.

Particle size remains a crucial parameter in determining the physicochemical properties associated with the IONP. The diameter of the nanoparticle is inversely proportional to the high-surface-to-volume ratio, causing smaller nanoparticles to have a larger surface area and more loading sites vacant for drug delivery and heavy metal removal applications [43]. Contrary to most extracellular IONP production by anaerobic bacteria which result in polydispersed nanoparticles with poor morphology, the IONP’s generated by MS2 show relatively high monodispersity as demonstrated by a low polydispersity index (Fig. 6) and narrow size distribution (30–60 nm).

The FT-IR spectrum of biosynthesized nanoparticles (Fig. 7), show weak banding in the region of 420–430 cm^−1^ due to the stretching of Fe–O bonds. These bands are of low intensity probably due to the extra bands between 1250 and 1390 cm^−1^ (Fig. 7a, b) that assigned to amines that may function as capping agents in the extracellular matrix to stabilize the biosynthesized IONP’s in colloidal solution.

## Conclusion

On conclusion, discovering microbial nanofactories that have the potential for large scale generation of IONP’s are necessary to unravel alternative pathways in nanoparticle production that are environmentally sustainable, cost effective and less convoluted. In this work, MS2, a potential nanofactory was isolated that demonstrated the ability of rapid generation of IONP’s in its extracellular matrix when confronted with FeCL_3_·6H_2_O and FeSO_4_·7H_2_O as precursors in its supernatant. The ability to reduce ferric ions in solution could be attributed to the melanin like pigment which confers iron reductase activity to facilitate the uptake and assimilation of Fe ions. Characterization of the IONP’s using FESEM–EDX, UV Spectrophotometry, DLS and FTIR showed that these nanoparticles were relatively mono-dispersed, well defined octahedral crystals with narrow size distribution. These nanoparticles remained stable in colloidal solution for over a week, probably due to the capping carboxy-proteins present in its extracellular matrix.
